# Versatile Microfluidic Mixing Platform for High- and Low-Viscosity Liquids via Acoustic and Chemical Microbubbles

**DOI:** 10.3390/mi10120854

**Published:** 2019-12-05

**Authors:** Yanfang Guan, Baichuan Sun

**Affiliations:** School of Electromechanical Engineering, Henan University of Technology, Zhengzhou 450001, China; sunbaichuan1994@163.com

**Keywords:** microfluidic mixer, microbubble, zigzag microchannel, high viscosity

## Abstract

Microfluidic mixers have been extensively studied due to their wide application in various fields, including clinical diagnosis and chemical research. In this paper, we demonstrate a mixing platform that can be used for low- and high-viscosity liquid mixing by integrating passive (utilizing the special circulating crossflow characteristics of a zigzag microstructure and cavitation surfaces at the zigzag corners) and active (adding an acoustic field to produce oscillating microbubbles) mixing methods. By exploring the relationship between the active and passive mixing methods, it was found that the microbubbles were more likely generated at the corners of the zigzag microchannel and achieved the best mixing efficiency with the acoustically generated microbubbles (compared with the straight channel). In addition, a higher mixing effect was achieved when the microchannel corner angle and frequency were 60° and 75 kHz, respectively. Meanwhile, the device also achieved an excellent mixing effect for high-viscosity fluids, such as glycerol (its viscosity was approximately 1000 times that of deionized (DI) water at 25 °C). The mixing time was less than 1 s, and the mixing efficiency was 0.95 in the experiment. Furthermore, a new microbubble generation method was demonstrated based on chemical reactions. A higher mixing efficiency (0.97) was achieved by combining the chemical and acoustic microbubble methods, which provides a new direction for future applications and is suitable for the needs of lab-on-a-chip (LOC) systems and point-of-care testing (POCT).

## 1. Introduction

Microfluidic technology has been developed and applied in many fields in recent years, such as environmental detection [1,2,3], cell biology [4,5,6], and protein detection [7,8,9]. Moreover, microfluidic mixers play an important role in these fields. For instance, to study the role of the biological macromolecules in cells, it is necessary to mix the substrates with enzymes or proteins and use high-viscosity fluids to dissolve them before enzymatic reactions occur [10]. The efficient mixing of highly viscous fluids plays a significant role in many fields, such as clinical diagnosis, clinical synthesis [11,12,13,14], and biochemical reactions [15,16,17]. In particular, in clinical diagnosis, highly viscous liquids such as plasma [11] and sputum [12,13] must be mixed with chemical reagents or buffers for further detection. However, in the microfluidic flow process, because of the low Reynolds number (Re), the mixing usually depends on the diffusion between molecules, and the mixing efficiency is considerably low [18,19]. Therefore, the efficient mixing in microfluidic chips and developing a versatile mixing platform are important challenges.

The micromixing methods in microfluidic chips can be divided into two types: passive mixing [20,21,22,23,24] and active mixing [25,26,27,28,29,30]. Passive mixing methods mainly rely on changing the geometries of microchannels to interfere with the fluid flow conditions, thereby enhancing diffusion and forming chaotic advection. For example, Nguyen et al. [20] combined hydrodynamic focusing (reducing the transversal mixing path) and time-interleaved segmentation (shortening the axial mixing path) to enhance the mixing efficiency. Ren et al. [24] improved the mixing effect by exploring the flow and mixing of experimental and numerically simulated fluids in zigzag channels. Passive mixing devices have the advantages of operating without external driving devices and only requiring simple operations, which improve the mixing efficiency. There are still many disadvantages, such as the complex processing and sample residue that may form due to the complicated patterns or long channels.

Compared to passive mixing methods, active mixing methods overcome these disadvantages. Added external parts are used to enhance the mixing performance to otherwise simple microchannel structures. Active mixing usually combines thermal [25], pressure [26], optical [27], magnetic [28], electro-kinetic [29], and acoustic [30,31,32,33,34,35,36,37] methods to improve the mixing efficiency by changing the fluid performance and flow state. Among these methods, the use of acoustic waves to interfere with the fluid mixing is widely used because it does not contaminate the samples, it is highly efficient, and the operation is simple. Wang et al. [31] realized the mixing of a high-viscosity fluid (glycerol) using acoustic-transducer-generated microbubbles, and the mixing time continued for 2–4 s. Yang et al. [32] utilized the ultrasonic characteristics of piezoelectric transducers (PZT, with 60 kHz and 50 Vpp, where Vpp means the peak-to-peak voltage) to enhance the mixing of ethanol and water. Ahmed et al. [33] proposed a “horseshoe” structure in the microchannel combined with acoustic waves to capture the microbubbles and enhance the mixing effect. However, these devices either require long times to achieve mixing and/or involve complicated manufacturing processes, such as the horseshoe microchannel, which is not easy to fabricate.

In this paper, a passive mixing method (by changing the zigzag microchannel structural parameters) and an active mixing method (acoustic microbubbles generated by PZT) were combined to influence the mixing for low- and high-viscosity fluids by the cavitation of microbubbles. There have been many studies on microfluidic mixers that combine active and passive mixing [37], such as the combination of a magnetic field and serpentine channel [38], the combination of an electric field and channel with a curved pattern [39], and vortex-enhanced mixing by adding an incompressible axisymmetric jet [40]. However, to the best of our knowledge, the influence of bubbles generated by an acoustic field based on a zigzag structure and the effect of their combination has not been studied before. By exploring different shapes of zigzag microchannels and the driving conditions of PZT combination, the optimal parameters for enhancing the mixing effect in the acoustic field were found. Moreover, the wavy surface microstructure of the polydimethylsiloxane (PDMS) sidewall microchannel fabricated by soft lithography technology on a silicon mold could incept and cavitate the microbubbles. Deionized (DI) water and glycerol solutions were used as the research objects in the experiment to explore the effect of viscosity on the mixing and the device’s application potential in other fields. The results showed that the hybrid mixing method not only realized the mixing of DI water (viscosity: 0.8937 mPa·s, 25 °C) but also achieved the mixing of high-viscosity fluids (glycerol, viscosity: 800 mPa·s, 25 °C). A new microbubble generation method via a chemical reaction was also proposed in addition to the aforementioned method using acoustic waves and passing the gas into the channel through an external device [41], which is often complex and has difficulty in meeting the needs of point-of-care testing (POCT). Therefore, the simplicity, ease of operation, and cost of the devices should also be taken into account. In our experiments, the use of a chemical reaction to generate bubbles also enhanced the mixing efficiency. This method eliminated the need for complex external devices, complex operations, and professional operators, which can broaden its usage for on-site testing and use in economically underdeveloped areas. Furthermore, the mixing effect was further optimized using an acoustic field. The mixing method proposed in this paper, which combined an acoustic field and zigzag structure to mix the fluids, can be used in low-Reynolds-number fluid mixing and is suitable for more applications.

## 2. Materials and Methods

### 2.1. Materials

A piezoelectric transducer (PZT-3000 kHz, H4P163000, Fukuda Ultrasound Co., Ltd., Guangzhou, China), polydimethylsiloxane (PDMS, Sylgard 184, Dow-Corning, Midland, MI, USA), a lithography machine (ABM/6/350/NUV/DCCD/M, ABM, New York, NY, USA), a positive photoresist (ma-N 400, Micro Resist, Berlin, Germany), a charge coupled device (CCD) and microscope (Obvious Ltd., Co., China), a syringe pump (LSP01, Longer, Baoding, China), a signal generator (DG1022, Rigol, Beijing, China), a power amplifier (2375, TEGAM, Geneva, OH, USA), dilute hydrochloric acid and sodium carbonate solutions (Thermo Fisher, Waltham, MA, USA), and glycerol (Zibo Hije Chemical Ltd., Co., China) were used in this study.

### 2.2. Fabrication of Microfluidic Chips

**Figure 1:**
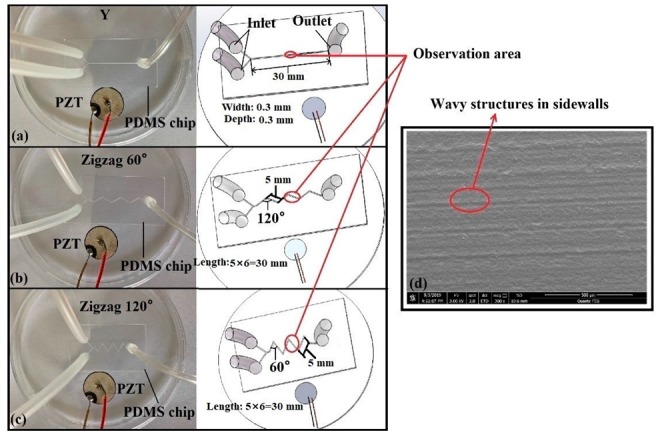
PDMS microfluidic chip: (**a**) Y-shaped (straight) microchannel, (**b**) zigzag microchannel with an angle of 120°, (**c**) zigzag microchannel with the angle of 60°, and (**d**) SEM image of the wavy microstructures of the microchannel sidewalls.

PDMS microfluidic chips were used in the experiment, and traditional lithography and replica molding technology [42,43,44] were used to fabricate the microchannel (the channel width and depth were 300 μm each, and the total length of the three chip channels was 30 mm) as shown in Figure 1a–c. First, a 4-inch silicon wafer was pretreated and coated with hexamethyldisilazane (HDMS) and a positive photoresist, respectively. Next, the silicon master mold was fabricated by soft lithography and deep reactive ion etching (DRIE) technology. After, the silicon mold was simply treated, and the PDMS (the ratio of the PDMS and curing agent was 10:1) was poured into the mold and cured. Finally, the PDMS device and a glass slide were bonded together by plasma treatment technology, and the required PDMS chip was obtained from the above steps. The wavy microstructures in the sidewalls of the silicon mold after DRIE are shown in Figure 1d. The rough surfaces could create voids in the flowing fluids, which promoted the generation of microbubbles under the action of acoustic waves.

### 2.3. Experimental Setup

As shown in Figure 1, to facilitate the generation of the microbubbles, the PZT was placed close to the PDMS microchip sidewalls, and they were fixed on the same plane. During the experiments, the driving voltage of the PZT was set to 100 V_pp_, and the frequencies were adjusted from 35 kHz to 2 MHz (35 kHz was the minimum frequency for bubble generation according to preliminary experiments). In addition, the acoustic field was applied after the fluid filled the microchannel to ensure that the flow of the fluids was steady. After, approximately 10 ms was required to generate the microbubbles using the applied ultrasound.

**Figure 2:**
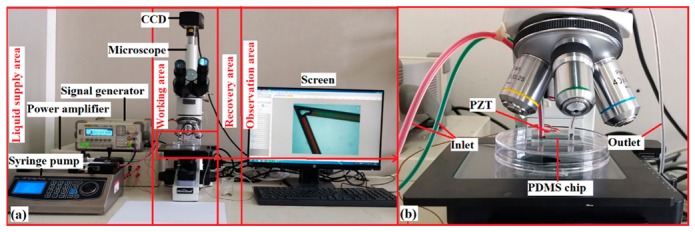
Mixing experiment platform: (**a**) Overall placement of mixing device and (**b**) Working area setup.

The experimental platform was divided into four parts: the liquid supply area, working area, recovery area, and observation area, as shown in Figure 2a. First, the prepared samples were injected into the microchannel from two inlets by a syringe pump simultaneously. The flow rate was set to 8 μL/min (the optimal speed to achieve stability and observability of the mixing based on five attempts). The samples met and mixed in the microchannel, as shown in Figure 2b. After, the mixed sample was recycled in the recovery area to avoid environmental pollution. The microbubble formation and mixing phenomenon inside the microchannel were observed under the microscope and were captured and photographed by the CCD. Finally, the image was grayscale processed using the ImageJ software (v1.8.0, National Institutes of Health, Bethesda, MD, USA) by converting the color images containing red, green, and blue (RGB) layers into grayscale values, after which the mixing index was calculated using Equation (1) (which represents the mixing effect between two kinds of fluids).

### 2.4. Mixing Index

To evaluate the mixing efficiency and better explain the mixing performance, the following mixing index equation was used [45,46]:

(1) $$M=1-\frac{\sqrt{1-n\sum {\left(I_{i}-I_{m}\right)}^{2}}}{I_{m}}$$

where M is the mixing index, n is the total number of points (pixel numbers in the selected areas of the images), $I_{i}$ is the intensity at each point, and $I_{m}$ is the average intensity. *M* = 1 corresponds to complete mixing between the fluids, and *M* = 0 denotes that the fluids did not mix at all. In addition, when the mixing index reaches 0.9 or higher (no greater than 1), it has been shown that excellent mixing occurs between fluids. Moreover, a mixing index in the 0.8–0.9 range indicates that the mixing is acceptable. However, when the mixing index is below 0.8, there is little mixing, or the mixing effect is unacceptable. 

## 3. Results and Discussion

### 3.1. Comparison of Mixing Efficiency with Different Microbubble Sizes

**Figure 3:**
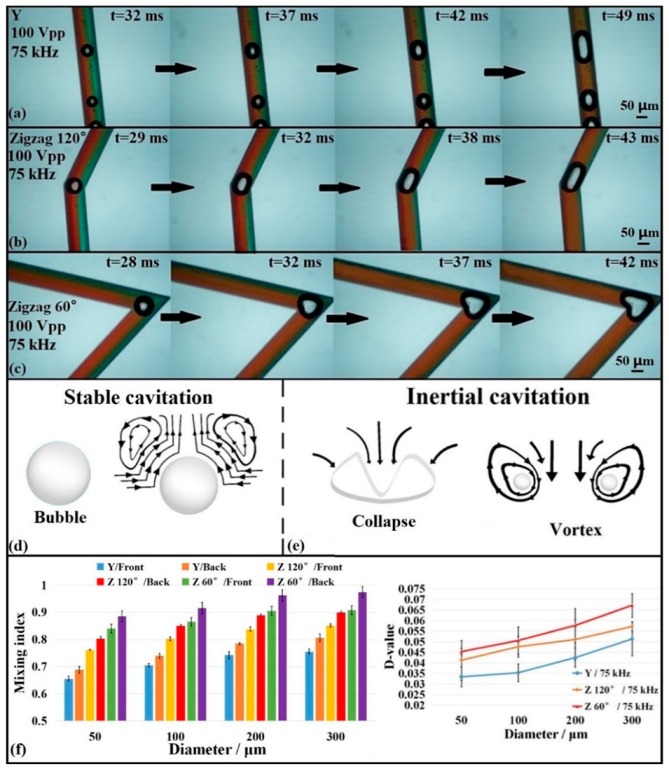
Comparison of microchannel structure vs. the microbubble and mixing efficiency: (**a**) Y-shaped microchannel, (**b**) zigzag microchannel with a 120° corner angle, (**c**) zigzag microchannel with a 60° corner angle, (**d**,**e**) schematic diagram of stable and inertial cavitation, and (**f**) comparison of the D-values between three kinds of microchannels. (n = 5, Mean ± SD).

To demonstrate that microbubbles affect mixing, DI water was mixed with red and green food dyes and injected into the microchannel at a flow rate of 8 μL/min separately. Figure 3a–c show that the sizes and generation locations of the microbubbles were different for the three microchannel structures under the action of the PZT (75 kHz and 100 V_pp_). The generation position of the microbubbles in the Y-channel was random, while for the zigzag channel, most of the bubbles were generated at the corners (a small number of bubbles were generated far away from the corner but close to the sidewalls). This occurred because the recirculating crossflow and the bend connecting the tilted channel segments in the zigzag channel broke down the laminar flow state of the fluids, and the roughness of the corners was higher (easier to produce bubbles) due to fabrication difficulties with the DRIE technology. Thus, we selected the half-length of the two zigzags (at the third corner) and Y-shaped microchannels as observation locations to ensure comparison consistency.

When the bubbles were generated by acoustic oscillations, cavitation occurred around the bubbles. Cavitation can be divided into two types: stable and inertial (or unstable) cavitation, according to the literature [47,48,49]. We drew a schematic diagram showing how bubbles influence the fluid flow during stable and inertial cavitation using AutoCAD software (2018, Autodesk Ltd., San Rafael, CA, USA), as shown in Figure 3d,e. The flow around the bubbles is disordered, which was one of the reasons the bubbles could interfere with the mixing as a result of these cavitation phenomena [50]. Stable cavitation creates small-scale vortices around the bubbles (Figure 3d); a large-scale vortex and jet will be generated around a small bubble after inertial cavitation. However, the inertial cavitation phenomenon that occurs under a high-intensity acoustic field (which is difficult to apply) will create high temperatures and pressure around the bubbles. Therefore, only stable cavitation occurred in our system, as we did not observe bubbles disappearing instantly (which indicated that there was no inertial cavitation) during the experiment based on the driving conditions used in this study. 

Different sizes of bubbles had different effects on the mixing, as shown in Figure 3a–c. For convenience, the mixing indices at the fronts and backs of the bubbles were calculated, and the difference value (*D-value* = *M_front_* − *M_back_*) was used as the mixing criterion to judge the influence of the bubble size. In addition, each bubble lasted about 15 ms from generation to departure, and new bubbles emerged at the same position immediately. As shown in Figure 3f, the D-value increased with the increase in the bubble size, which indicated that a higher mixing efficiency was obtained. Five samples were obtained under each set of driving conditions. (Note that in Figure 3 and all the followed figures, the error bars in the graphics represent the standard error, which was calculated based on the mean and standard deviation (SD) from the grayscale values for each experiment. Each experiment set in this paper was repeated five times (n = 5) and all the experimental datum were shown as Mean ± SD. Statistical analysis (P-value (*p*)) between contrast groups have been calculated using the one-way ANOVA method to observe the significance difference. *p* < 0.05 represents the significance difference.). This occurred because the laminar flow of the fluids in the microchannel was hindered by the enlarging microbubbles. When a microbubble size increased to the width of the microchannel, the flow in front and back of the microbubble was restricted. Consequently, the fluids mixed because of the cavitation, as shown in Figure 3d,e. Thus, the mixing efficiency was proportional to the microbubble size. The mixing time (mixing index was more than 0.8) was less than 1 s, as shown in Figure 3a–c because of the combination of the acoustic microbubbles and special zigzag microchannel structure. Furthermore, the driving frequency, voltage, and liquid viscosity had important influences on the microbubble size.

### 3.2. Comparison of Mixing Efficiency with Different Microchannel Structures

**Figure 4:**
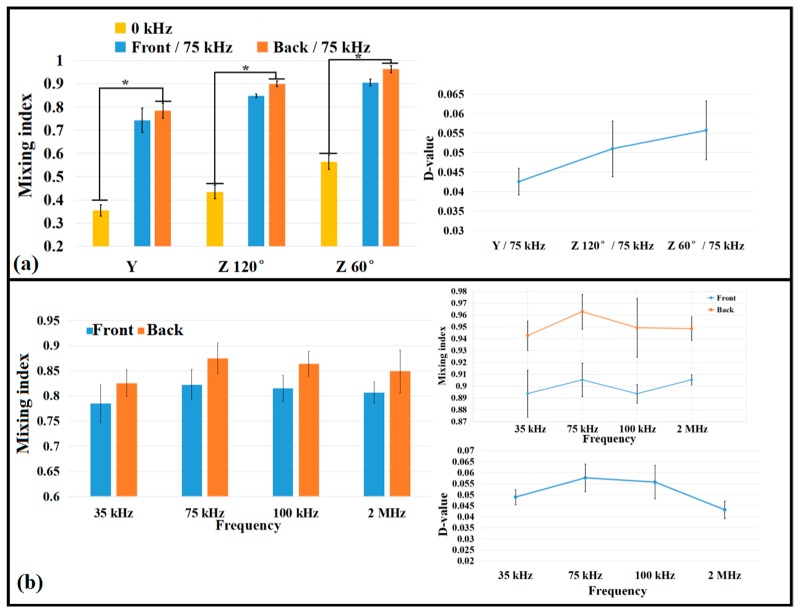
Comparison of mixing efficiencies under different microchannel structures and driving conditions. (**a**) Influence of the channel structures for the mixing index and (**b**) comparison the mixing results vs. frequency with a 60° corner angle. (n = 5, Mean ± SD, *: *p* < 0.05).

In addition to the influence of the microbubble size on fluid mixing, the microchannel structure also had an important influence, as shown in Figure 3a–c. The bubble shape, position, and time due to the acoustic actuation were different in the Y-shaped microchannel and the zigzag microchannels with 60° and 120° corner angles due to the differences of the microchannel structures. Based on the calculated mixing index, the fluid mixing with the zigzag structures was better than that in the straight microchannel, and the 60° corner angle yielded better mixing than the 120° corner angle, because the zigzag channel geometry optimized the mixing effect, as shown in Figure 4. The mixing index of the zigzag channel was significantly higher than that of the straight channel (when the PZT driving power was off; i.e., at 0 kHz) in Figure 4a. Oscillating bubbles (taking stable bubbles with diameters of about 150 μm as the object of study) were generated in the presence of the acoustic waves (75 kHz), which interfered with the fluid flow and mixing. Stable cavitation occurred with the generation or movement of the microbubbles (Figure 3d), which created counter-rotating vortices that interfered with the fluid flow, enhancing the mixing effect. During the fluid motion, the microbubbles did not collapse sufficiently to form inertial cavitation, and the time from when the bubble was generated to when it flowed away was about 15–20 ms. As shown in Figure 4, the mixing effect was significantly enhanced when the acoustic field was applied. The smaller the zigzag angle, the better the effect, which is consistent with the research of Tasi et al. [51]. The mixing effect was further optimized when the frequency increased to 75 kHz due to stable cavitation interactions, as shown in Figure 4a.

As depicted in Figure 4a, the mixing effect of the zigzag channel with a 60° corner angle was the best, regardless of whether there was an acoustic field. In this channel (Figure 4b), the acoustic field produced by the PZT with different frequencies also affected the mixing, which was consistent with the research of Orbay et al. [41] and Hashmi et al. [46]. Once the acoustic field was present, oscillating bubbles caused cavitation phenomena, and the acoustic surface wave acted simultaneously. The mixing effect changed with the frequencies, especially at 75 kHz, which yielded the best mixing performance, as shown in Figure 4b. As the driving efficiency was enhanced (higher than 75 kHz, the maximum value was 2 MHz in our experiment), the fluid mixing did not increase but decreased slightly. One explanation is that 75 kHz was the resonance frequency response point for the device. Under these driving conditions (75 kHz, 100 V_pp_), various sizes of microbubbles emerged, especially in back of the main bubbles, which may have enhanced the mixing effect (shown in Figure 3d,e) due to the cavitation of microbubbles. Moreover, bubbles inevitably collided during the movement process, and the bubble—bubble interactions produced a vortex flow according to the Rayleigh–Plesset theory [46]. All these effects enhanced the mixing efficiency.

### 3.3. Comparison of Mixing Effect with Different Fluid Viscosities 

**Figure 5:**
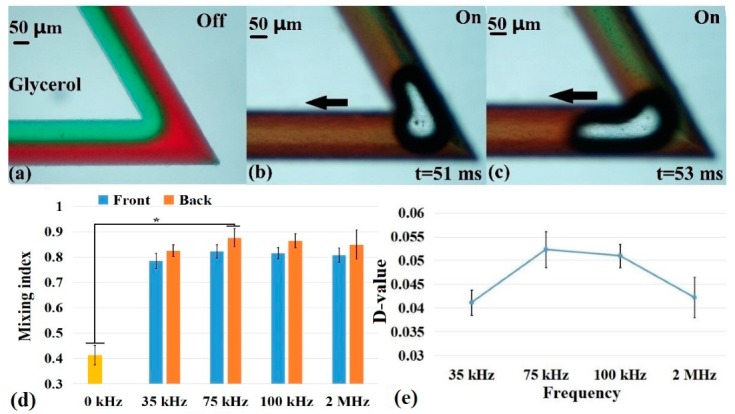
High-viscosity glycerol mixing conditions: (**a**) no mixing occurred when the acoustic field was powered off, (**b**,**c**) the microbubble motion at the corner of the zigzag microchannel when the acoustic field was turned on, (**d**) comparison of the mixing effect at the fronts and backs of the microbubbles under different driving frequencies, and (**e**) D-value comparison of the microbubbles under different driving frequencies. (n = 5, Mean ± SD, *: *p* < 0.05).

The viscosities of the liquids have a great influence on mixing and can be used in many applications, such as blood testing [43] and biochemical reactions [15]. Therefore, glycerol was used as the mixing sample (for easy observation, the glycerol was mixed with red and green food dyes, as shown in Figure 5a with a 60° corner angle in the zigzag microchannel) in our experiment to evaluate the mixing performance in the high-viscosity fluid with the PDMS chip. The viscosity of pure glycerol was approximately 1000 times that of DI water (at 25°C, the viscosity of DI water and glycerol were 0.8937 and 800 mPa·s, respectively). In Figure 5a, glycerol laminar flow occurred without the PZT driving (PZT power was off), which means that no mixing between the two fluids occurred without the acoustic field in the zigzag microchannel. However, when the acoustic field was turned on, the two glycerol solutions mixed with the emergence of a microbubble at the first corner under the action of the acoustic field oscillation and bubble cavitation, and the mixing effect was enhanced with the bubble flow (i.e., the black arrows in Figure 5b,c).

In addition, the maximum mixing index still occurred at 75 kHz for the glycerol solutions, followed by 100 kHz, which was similar to the results of DI water. However, the effect of glycerol mixing was significantly worse than the low-viscosity fluids based on the comparison of the mixing indices for DI water and glycerol shown in Figure 4b and Figure 5d. The mixing index of DI water (0.96) was larger than that of glycerol (0.86) under the same driving conditions (such as 75 kHz and 100 V_pp_). This occurred because the generation and growth of bubbles were also affected (the generation time was longer than that of the DI water) due to the high viscosity of the glycerol, which was related to the compression and expansion cycle of acoustic waves [36]. Although the viscosity of glycerol was very high, the mixing index at the outlet was 0.95, and the total mixing time was less than 1 s. By comparing the D-values between the fronts and backs of the microbubbles, it was found that, at 75 kHz, the D-value reached about 0.05, which was slightly smaller than that for the mixing of DI water, indicating that the influence of the bubbles and acoustic waves in the liquid with a high viscosity was worse than that of liquid with low viscosity.

### 3.4. Mixing Analysis via Chemical Reaction

**Figure 6:**
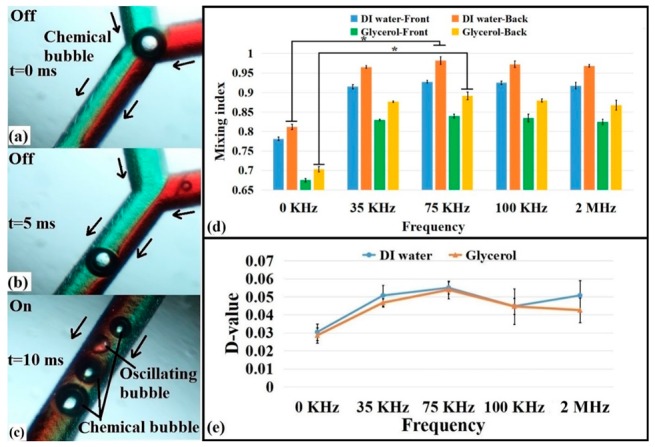
Diagram of the chemical reaction of microbubbles and the mixing efficiency analysis: (**a**) chemical microbubbles in a zigzag microchannel, (**b**) chemical bubbles flow with the fluid flow, (**c**) chemical bubbles and oscillating bubbles coexist when the piezoelectric transducer (PZT) was turned on, (**d**) comparison of mixing indices with different viscosities fluids with chemical and acoustic microbubbles, and (**e**) comparison of the D-values under different driving frequencies. (n = 5, Mean ± SD *: *p* < 0.05).

The microbubbles can also be generated by chemical reactions in addition to generating microbubbles acoustically and injecting gas to generate microbubbles using external devices. To demonstrate this, a dilute hydrochloric acid solution (10% of the mass fraction with DI water or glycerol) and sodium carbonate solution (10% of the mass fraction with DI or glycerol) were mixed in a ratio of 2:1 to produce microbubbles. Two kinds of solutions including microbubbles (generated in advance) were injected into the microchannel using a syringe pump, as shown in Figure 6a,b. The distinct advantage of this method was that the microbubbles generated by the chemical reaction were used to influence the fluid mixing without the presence of an acoustic field, which simplifies the devices required. Once the acoustic field was present, there were not only the chemical bubbles in the channel but also the oscillating bubbles generated by acoustic vibrations, as shown in Figure 6c. Thus, the coexistence of two kinds of microbubbles strengthened the conductivity of the fluidics.

The chemical bubbles themselves also affected the mixing (at 0 kHz), as depicted in Figure 6d,e, but the results were smaller than those in the presence of an acoustic field (the driving frequency was from 35 kHz to 2 MHz). Moreover, these results were compared to those for the acoustic field alone shown above (Figure 4b). The results for the system with only the acoustic field were better than those with only the chemical reaction. This further proved that the presence of an acoustic field influenced the cavitation effect around the bubbles. The acoustic field still played a role in enhancing the mixing effect, but the pre-made chemically induced microbubbles in the system were an ideal control scheme because there was no need to add more external devices, which is a good method to reduce costs and save space. In addition, after adding chemical bubbles, there were more bubbles in the channel, which was one of the reasons this method could increase the mixing effect. A comparison of the mixing indices and D-values in the microchannel after adding the chemical bubbles showed that the mixing results were better than those when only acoustic waves were used. The mixing efficiency of deionized water and glycerol reached almost 1 and 0.97, respectively. However, there were still some shortcomings of the chemical method, such as the need to find chemical reagents that can generate bubbles and ensuring that the samples were not affected by these reagents. Despite these limitations, this approach is feasible and efficient.

## 4. Conclusions

In conclusion, we demonstrated the superiority and feasibility of the combination of a passive mixer (zigzag structure) and an active mixer (bubbles generation by an acoustic field). In the presence of an acoustic field, the mixing effect was the best when the corner angle of the zigzag channel was 60°. The bubbles were more likely to appear at the corners of the channel, which eliminated the random appearance of bubbles that occurred in straight channels. Meanwhile, different frequencies were applied to explore the influence of different acoustic fields on mixing, and better results were achieved when the frequency was 75 kHz. More importantly, the mixer also realized the mixing of high-viscosity fluids (pure glycerol, viscosity was approximately 1000 times that of DI water at 25 °C). The mixing time was less than 1 s, and the mixing efficiency was about 0.95. In addition, we proposed a new method for generating bubbles using a chemical reaction, which eliminated the need for external equipment to generate bubbles and made the device more convenient to use. Moreover, combined with the effect of the acoustic field, more efficient mixing was achieved; the final mixing efficiency of glycerol reached 0.97. In this way, the mixing was better under the action of both types of bubbles—chemical and acoustic. However, there are still limitations to our present study. Appropriate chemical reagents must be selected to react and generate bubbles, and the sample must not be affected by these reagents. Moreover, more components or an additional outlet at the end of the microchannel will be added to remove the bubbles in our future research, because the presence of the microbubbles is harmful to the fluid system or analytical process. Nevertheless, the use of acoustic waves to generate oscillating bubbles in microfluidic mixers shows potential because of its environmental friendliness and low cost. 

## References

[1] Deng S., Yang T., Zhang W., Ren C., Zhang J., Zhang Y., Cui T., Yue W. (2019). Rapid detection of trichlorfon residues by microfluidic paperbased phosphorus-detection chip (μPPC). N. J. Chem..

[2] Maruyama H., Otake T., Arai F. (2011). Photoprocessible hydrogel microsensor for local environment measurement on a microfluidic chip. IEEE ASME Trans. Mechatron..

[3] Digiacomo L., Palchetti S., Giulimondi F., Pozzi D., Chiozzi R.Z., Capriotti A.L., Lagna A., Caracciolo G. (2019). The biomolecular corona of gold nanoparticles in a controlled microfluidic environment. Lab Chip.

[4] Soitu C., Feuerborn A., Tan A.N., Walker H., Walsh P.A., Castrejón-Pita A.A., Cook P.R., Walsh E.J. (2018). Microfluidic chambers using fluid walls for cell biology. Proc. Natl. Acad. Sci. USA.

[5] Babic J., Griscom L., Cramer J., Coudreuse D. (2018). An easy-to-build and re-usable microfluidic system for live-cell imaging. BMC Cell Biol..

[6] Li C., Li W., Geng C., Ren H., Yu X., Liu B. (2018). Microfluidic chip for cancer cell detection and diagnosis. J. Mech. Med. Biol..

[7] Fraas R., Hübner J.F., Diehm J., Faas R., Hausmann R., Franzreb M. (2019). A Compartmented microfluidic reactor for protein modification via solid-phase reactions—Semi-automated examination of two PEGylation routes. Biotechnol. Bioprocess. Eng..

[8] Lin Y.-H., Chang H.-Y., Wu C.-C., Wu C.-W., Chang K.-P., Yu J.-S. (2019). BRAF protein immunoprecipitation, elution, and digestion from cell extract using a microfluidic mixer for mutant BRAF protein quantification by mass spectrometry. Anal. Bioanal. Chem..

[9] Sinkala E., Sollier-Christen E., Renier C., Rosas-Canyelles E., Che J., Heirich K., Duncombe T.A., Vlassakis J., Yamauchi K.A., Huang H. (2017). Profiling protein expression in circulating tumour cells using microfluidic western blotting. Nat. Commun..

[10] Gorkin R., Park J., Siegrist J., Amasia M., Lee B.S., Park J.-M., Kim J., Kim H., Madou M., Cho Y.-K. (2010). Centrifugal microfluidics for biomedical applications. Lab Chip.

[11] Thom J., Lvey L., Eikelboom J. (2003). Normal plasma mixing studies in the laboratory diagnosis of lupus anticoagulant. J. Thromb. Haemost..

[12] Xie Y., Todd N.W., Liu Z., Zhan M., Fang H., Peng H., Alattar M., Deepak J., Stas S.A., Jiang F. (2010). Altered miRNA expression in sputum for diagnosis of non-small cell lung cancer. Lung Cancer.

[13] Wu J., Hillier C., Komenda P., de Faria R.L., Santos S., Levin D., Zhang M., Lin F. (2016). An all-on-chip method for testing neutrophil chemotaxis induced by fMLP and COPD patient’s sputum. Technology.

[14] Yang S., Guo F., Kiraly B., Lu M., Leong K.W., Huang T.J. (2012). Microfluidic synthesis of multifunctional Janus particles for biomedical applications. Lab Chip.

[15] Schabas G., Yusuf H., Moffitt M.G., Sinton D. (2008). Controlled self-assembly of quantum dots and block copolymers in a microfluidic device. Langmuir ACS J. Surf. Colloids.

[16] Gao D., Jin F., Zhou M., Jiang Y. (2019). Recent advances in single cell manipulation and biochemical analysis on microfluidics. Analyst.

[17] Lam R.H., Cui X., Guo W., Thorsen T. (2016). High-throughput dental biofilm growth analysis for multiparametric microenvironmental biochemical conditions using microfluidics. Lab Chip.

[18] Mao X., Huang T.J. (2012). Microfluidic diagnostics for the developing world. Lab Chip.

[19] Mao X., Waldeisen J.R., Huang T.J. (2007). “Microfluidic drifting”—Implementing three-dimensional hydrodynamic focusing with a single-layer planar microfluidic device. Lab Chip.

[20] Nguyen N.-T., Huang X. (2005). Mixing in microchannels based on hydrodynamic focusing and time-interleaved segmentation: Modelling and experiment. Lab Chip.

[21] Schonfeld F., Hessel F., Hofmann C. (2004). An optimised split-and-recombine micro-mixer with uniform ‘chaotic’ mixing. Lab Chip.

[22] Kim D.S., Lee S.H., Kwon T.H., Ahn C.H. (2005). A serpentine laminating micromixer combining splitting/recombination and advection. Lab Chip.

[23] Xia H.M., Wang Z.P., Koh Y.K., May K.T. (2010). A microfluidic mixer with self-excited ‘turbulent’ fluid motion for wide viscosity ratio applications. Lab Chip.

[24] Ren Y., Leung W.W. (2013). Flow and mixing in rotating zigzag microchannel. Chem. Eng. J..

[25] Tsai J.H., Lin L. (2002). Active microfluidic mixer and gas bubble filter driven by thermal bubble micropump. Sens. Actuators A Phys..

[26] Glasgow I., Aubry N. (2003). Enhancement of microfluidic mixing using time pulsing. Lab Chip.

[27] Hellman A.N., Rau K.R., Yoon H.H., Bae S., Palmer J.F., Phillips K.S. (2007). Laser-induced mixing in microfluidic channels. Anal. Chem..

[28] Bau H.H., Zhong J., Yi M. (2001). A minute magneto hydro dynamic (MHD) mixer. Sens. Actuators B Chem..

[29] Chang C.-C., Yang R.-J. (2007). Electrokinetic mixing in microfluidic systems. Microfluid. Nanofluid..

[30] Phan H.V., Coskun M.B., Sesen M., Pandraud G., Neild A., Alan T. (2015). Vibrating membrane with discontinuities for rapid and efficient microfluidic mixing. Lab Chip.

[31] Wang S., Huang X., Yang C. (2011). Mixing enhancement for high viscous fluids in a microfluidic chamber. Lab Chip.

[32] Yang Z., Goto H., Matsumoto M., Maeda R. (2000). Active micromixer for microfluidic systems using lead-zirconate-titanate (PZT)-generated ultrasonic vibration. Electrophoresis.

[33] Ahmed D., Mao X., Shi J., Juluri B.K., Huang T.J. (2009). A millisecond micromixer via single-bubble-based acoustic streaming. Lab Chip.

[34] Liu R.H., Yang J., Pindera M.Z., Athavale M., Grodzinski P. (2002). Bubble-induced acoustic micromixing. Lab Chip.

[35] Brotchie A., Grieser F., Ashokkumar M. (2009). Effect of power and frequency on bubble-size distributions in acoustic cavitation. Phys. Rev. Lett..

[36] Ozcelik A., Ahmed D., Xie Y., Nama N., Qu Z., Nawaz A.A., Huang T.J. (2014). An acoustofluidic micromixer via bubble inception and cavitation from microchannel sidewalls. Anal. Chem..

[37] Hessel V., Löwe H., Schönfeld F. (2005). Micromixers—A review on passive and active mixing principles. Chem. Eng. Sci..

[38] Meijer H.E.H., Singh M.K., Kang T.G., den Toonder J.M.J., Anderson P.D. (2009). Passive and active mixing in microfluidic devices. Macromol. Symp..

[39] Kunti G., Bhattacharya A., Chakraborty S. (2017). Rapid mixing with high-throughput in a semi-active semi-passive micromixer. Electrophoresis.

[40] Depuru Mohan N.K., Greenblatt D., Nayeri C.N., Paschereit C.O., Panchapakesan N.R. (2015). Vortex-enhanced mixing through active and passive flow control methods. Exp. Fluids.

[41] Orbay S., Ozcelik A., Lata J., Kaynak M., Xu M., Huang T.J. (2017). Mixing high-viscosity fluids via acoustically driven bubbles. J. Micromech. Microeng..

[42] Enert D., Bhushan B. (2016). Transparent, superhydrophobic, and wear-resistant surfaces using deep reactive ion etching on PDMS substrates. J. Colloid Interface Sci..

[43] Zhang X.-S., Chu S.-G., Peter N., Zhang H.-X. Fabrication of tunable wetting PDMS membrane by nanostructuring and plasma treatment. Proceedings of the 8th Annual IEEE International Conference on Nano/Micro Engineered and Molecular Systems.

[44] Raimbault V., Rebiere D., Dejous C., Guirardel M., Conedera V. (2008). Acoustic Love wave platform with PDMS microflfluidic chip. Sens. Actuators A Phys..

[45] Li Y., Xu Y., Feng X., Liu B.-F. (2012). A rapid microfluidic mixer for high-viscosity fluids to track ultrafast early folding kinetics of G-quadruplex under molecular crowding conditions. Anal. Chem..

[46] Hashmi A., Xu J. (2014). On the quantification of mixing in microfluidics. J. Lab. Autom..

[47] Zwaan E., Le Gac S., Tsuji K., Ohl C.D. (2007). Controlled cavitation in microfluidic systems. Phys. Rev. Lett..

[48] Versluis M. (2000). How snapping shrimp snap: Through cavitating bubbles. Science.

[49] Hashmi A., Yu G., Reilly-Collette M., Heiman G., Xu J. (2012). Oscillating bubbles: A versatile tool for lab on a chip applications. Lab Chip.

[50] Brennen C.E. (1995). Cavitation and Bubble Dynamics.

[51] Tsai C.-H.D., Lin X.-Y. (2019). Experimental study on microfluidic mixing with different zigzag angles. Micromachines.

